# Daymet and DeGAUSS: Development of a Novel Method that Links High Spatial Resolution Gridded Meteorological Data with Geocoded Healthcare Encounters

**DOI:** 10.23889/ijpds.v9i5.1672

**Published:** 2024-09-18

**Authors:** Benjamin Barrett, Peter Graffy, Daniel Horton, Abel Kho

**Affiliations:** 1 Northwestern University

## Objective

Due to human-caused climate change, extreme temperature-related morbidity/mortality are increasing global public health concerns. To better understand the burdens of heat exposure, researchers must merge accurate/granular environmental data with clinical data. We developed a privacy-preserving method that links high-spatial-resolution continuous, gridded, meteorological data to geocoded healthcare encounters.

## Approach

In an R environment, we developed code that links geocoded healthcare encounters to publicly-available Daymet data via the corresponding Daymet raster cell number and healthcare encounter date. Daymet provides daily 1km x 1km surface meteorological data over North America since 1980. User specifications include the bounding box latitude/longitude of Daymet data download, which Daymet environmental variables are linked, and the day-lag of Daymet data matching to healthcare encounter date.

## Results

Linking 500,000 healthcare encounters over a 7-day lag with 7 years of Daymet data comprising minimum/maximum temperature over a bounding box encompassing Cook County, Illinois (5,670 1km raster cells) took 11.92 minutes with 4 CPUs and 8GB RAM. Thirteen years of Daymet data took 23.85 minutes. Linking 7.5 million encounters with 7 years of data took 28.49 minutes.

## Conclusion

Our newly-developed method for linking Daymet data to geocoded healthcare encounters preserves patient privacy, and processes the data efficiently over a minimal timespan.

## Implications

Daymet data linked to healthcare encounters allows for accurate assessment of the association between environmental variable exposure and any clinical outcome over North America. Packaging the code allows for public distribution within the Decentralized Geomarker Assessment for Multi-Site Studies (DeGAUSS) universe of geoanalytic tools.

